# The influence of characteristic scales of convection on non-isothermal evaporation of a thin liquid layer

**DOI:** 10.1038/s41598-018-29015-3

**Published:** 2018-08-01

**Authors:** S. Y. Misyura

**Affiliations:** 1 National Research Tomsk Polytechnic University, pr. Lenina 30, Tomsk, 634050 Russia; 2 Institute of Thermophysics Siberian Branch, Russian Academy of Sciences, Lavrentiev Ave. 1, Novosibirsk, 630090 Russia

## Abstract

Here, the effect of convection in liquid on non-isothermal evaporation of a horizontal thin layer on a hot wall is investigated. It is considered that the evaporation rate of salts always decreases with the growth of salt concentration. Depending on the nature of evaporation rate, the aqueous salt solutions can be classified into two different types: (1) the equilibrium partial pressure of water vapor *p*_*s*_ varies slightly with time; (2) with an increase in salt mass concentration, *p*_*s*_ decreases many times, which leads to a sharp drop in evaporation rate *j*. The criteria for attributing the salt to characteristic types are proposed, and relation between *j* and thermodynamic properties of salt solutions is determined. Different approaches to modeling are proposed for each group. For the first time, a simple calculation method linking the Peclet and Marangoni criteria with convection in a liquid and non-stationary heat exchange is proposed. The analysis shows that it is impossible to simulate the heat transfer without knowing the local characteristics of the velocity field in the liquid phase and without clearly distinguishing the characteristic convective scales of the velocity and temperature fields. So far, it has been believed that the surface Marangoni flow can be neglected due to the negative impact of surfactants. However, the studies of this paper show that a noticeable increase in free convection relates to the thermal and solutal Marangoni flows. A strong influence of the Marangoni flow on liquid convection at high heat fluxes is extremely important for reliable simulation of layer evaporation in a wide range of modern technologies.

## Introduction

Aqueous salt solutions represent an important field of research that links a wide range of different disciplines: thermodynamics of solutions, physical chemistry of the phase boundary, crystallography, kinetics of crystallization, hydrodynamics and heat exchange in a thin layer of a solution. Heat and mass transfer in solutions cannot be modeled taking into account only the transfer of heat, momentum and energy in the bulk phase of the liquid. No less important is the role played by surface flows on the boundary of liquid-gas. The small size of the film significantly limits the experimental possibilities and complicates the theoretical analysis.

During evaporation of water-salt solutions, local supersaturation regions may occur, where crystals and crystallohydrates of different morphology appear^1^. Absorption and evaporation of salt solutions: LiCl/H_2_O, LiBr/H_2_O and CaCl_2_/H_2_O, are effectively used by the modern heat pumps^2^.

The following factors determine the character of salt solution behavior: gas pressure^3^, concentrations of components and temperatures at the free liquid surface, thermophysical properties of a liquid and a wall^4^, wettability, liquid and gas convection^5^. Free convection and turbulence degree in the gas phase intensify the transfer processes^6^.

Since heat transfer depends on the film thickness, it is of interest to study the stability of the film. The film instability is driven by disjoining pressure. Resonant interaction between the interfacial deformations and the substrate structuring pattern leads to discontinuities in the dispersion curves, a situation analogous to appearance of gaps in the energy spectra seen in the applications of Floquet theory in solid state physics^7^.

When the liquid film spreads over a hot wall having an uneven temperature distribution, three-dimensional circulations inside the liquid, a change in direction in the liquid and a local rupture of the film occur^7^. This unstable behavior of the film is caused by the thermal Marangoni flow. The localized thermocapillary flow and deformation of the gas-liquid interface happen at laser cutting of metals^8^, when nanoparticles are deposited from the solution by laser radiation^9^. Features of thermocapillary forces in a thin film are also considered in^10,11^. Heat exchange at evaporation of films is considered in^10,11^. Heat transfer at film evaporation was discussed in^12,13^.

Modeling heat transfer of a salt solution is fundamentally different from modeling heat transfer in a water layer. The physical and thermodynamic properties of solution (latent heat of evaporation, diffusion coefficient, viscosity, surface tension) vary markedly with increasing salt concentration in the solution^14^. The density of evaporation flux of aqueous salt solutions can fall many times with time.

In most numerical studies modeling of the evaporation rate of a film does not take into account free convection of gas and liquid^15–17^. Simulation of heat exchange in lubricant emulsions is presented in^18^. Physical properties of aqueous salt solutions are presented in^19–21^. A membrane desorber and condenser with salt solution are studied experimentally in^22^. Features of the behavior of the heat transfer coefficient of the falling film of mixtures are considered in^23^.

Based on the analysis of the existing literature, the following conclusions may be drawn. Most experimental and theoretical works on absorption and evaporation dealt with LiCl, CaCl_2_ and LiCl salts. These salts are often used in the high-temperature generators of heat pumps. There are scarce experimental data on non-isothermal heat exchange of films at multiple changes in the layer height and salt concentration. Our previous research works concerned non-isothermal evaporation of large drops of water-salt solutions. Droplets with large diameters (*d* > 20–30 mm) have a quasi-plane shape and can therefore partly reflect the behavior of the thin layer. However, the simulation of large drops and thin liquid layers is principally different. In the drop, high temperature gradient *T*_*s*_ occurs on the interface. In addition, in the vicinity of a contact line there is a sharp change in the solution height and a high gradient of *j*. For a layer, the liquid height does not depend on the longitudinal coordinate, and simulating the surface temperature gradient is difficult. In addition, there experimental data on non-isothermal high-temperature evaporation and heat exchange of thin layers are very scarce. Most of the works were performed at a fixed salt concentration, viscosity, diffusion and liquid film height. These limitations do not allow accurately reproducing and modeling modern technologies dealing with high-temperature saline solutions.

In connection with the above, one of the objectives of this work is to obtain experimental and theoretical dependences for the evaporation rate and heat transfer coefficient, when the thermophysical properties of the salt solution and the height of the layer during evaporation change many times.

To date there is no comprehensive investigation of non-isothermal heat exchange for a large number of salts, whose thermal properties differ significantly. As a rule, equilibrium curves and thermophysical parameters of salts are analyzed in the literature. Another important goal of this work is to identify the key parameters by which the salt can be attributed to the characteristic group. It is also crucial to determine how the evaporation rate and heat transfer coefficient are related to the properties of salts; to obtain qualitative and quantitative dependences for the behavior of highly concentrated solutions, which will help to further develop the existing calculation models.

Another purpose of the research is to determine the role of the Marangoni flow at a layer surface. Currently, it is widely believed that the role of the Marangoni number on the liquid evaporation due to the influence of surfactant and the role of convection in the thin layer are insignificant. However, these approximations are not always fair. Convection and Marangoni flow not only in a drop, but also in a thin layer of the solution have an important role in the heat and mass transfer.

## Measuring Technique

Two-component water/salt solutions were used in all experiments: CsCl; NaCl; BaCl_2_; CaCl_2_; LiBr; LiCl and MgCl_2_. Evaporation behavior of these salts differs significantly, and for convenience of their description and modeling, the aqueous salt solutions can be divided into two types. The first type with a quasi-constant *j* includes salts: CsCl; NaCl and BaCl_2_. For the second type of salts (LiCl; CaCl_2_; LiBr, and MgCl_2_), *j* becomes several times lower. The reason for such different behaviors will be considered in the next section. The initial height of the aqueous salt solution *δ*_0_ and the initial salt concentration *С*_01_ were the same for all experiments (*δ*_0_ = 2.95–3 mm, *С*_01_ = 9–10%). The initial height of the layer was set at initial mass *m*_0_, which was calculated in advance. In this case, the initial height was determined as follows *δ*_0_ = *m*_0_/*ρF*_0_, where *F*_0_ = π*R*^2^, *R* is the radius of the heater, *ρ* is the density of the liquid (water or ten percent salt solution, which was determined by the densimeter).

**Figure 1:**
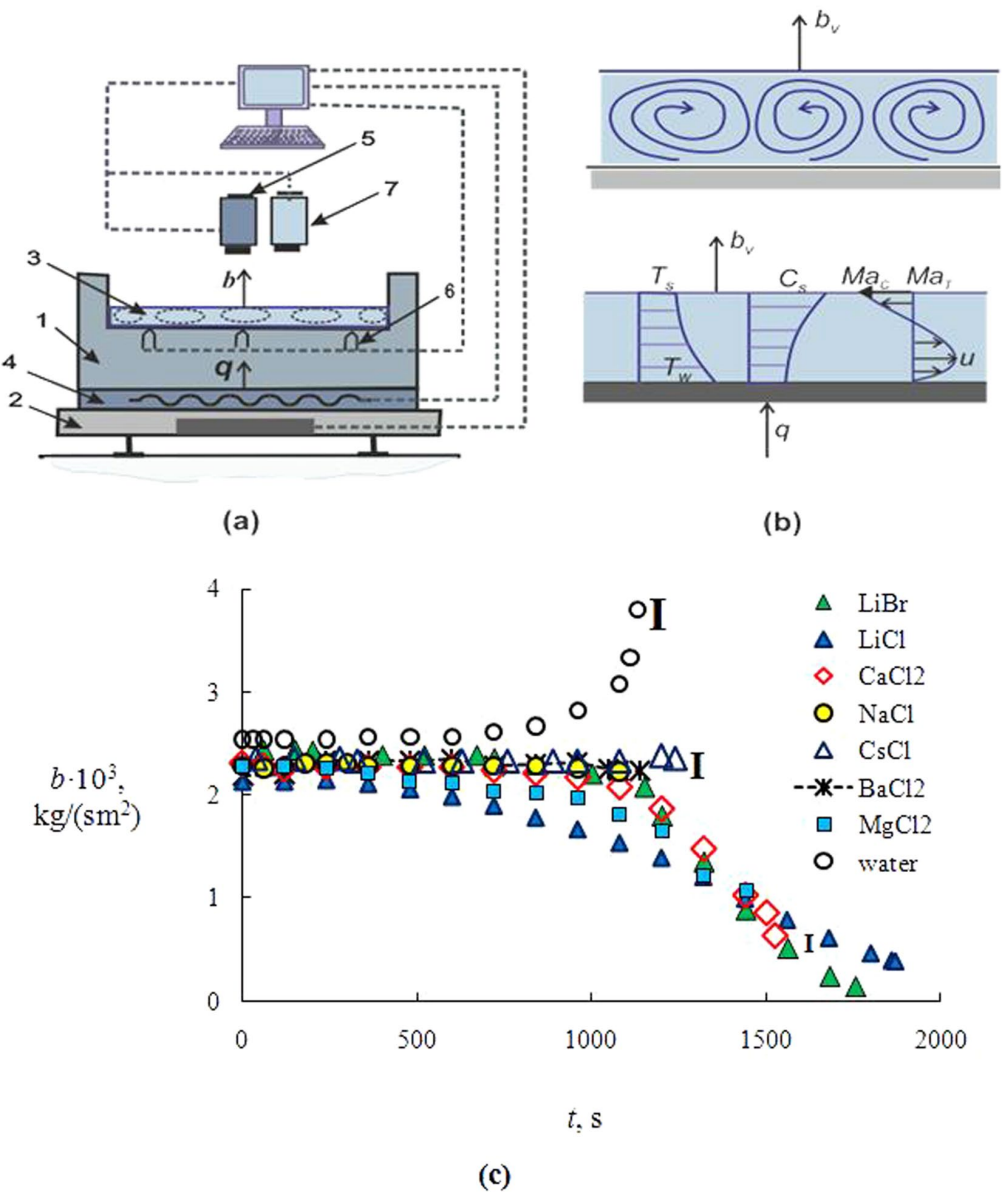
(**a**) The scheme of setup: 1 – working metallic section (titanium); 2 – electronic balance; 3 – liquid layer; 4 – electric heater; 5 – NEC R500; 6 – thermocouples; 7 – video camera; (**b**) the distribution profiles of temperature, salt concentration and liquid velocity inside the solution layer; (**c**) evaporation rate *b* (kg/(cm^2^)) of liquid layer (*С*_01_ = 10%; *T*_w_ = 80 °C); **I** is the interval of measurement errors.

The ambient temperature ranged from 22–24 °C, and the relative air humidity was varied in the range of 35–40%. To maintain quasi-constant humidity of outdoor air, silica gel was used, and the humidity was controlled by the humidity meter. All experiments were carried out at atmospheric pressure of air of 1 bar. The scheme of the working setup is shown in Fig. 1(a). The working section of the setup (1) was made of titanium (diameter *d* = 70 mm). The use of titanium virtually eliminated corrosion due to the salt and ensured the constant wettability. Measurements of the contact angle of the drop before and after the experiments showed a satisfactory convergence of 57 ± 3°. Solutions of salts (3) were poured on the heated site (1). Heating was carried out by means of a heater (4). Inside the heater there was an evenly distributed tungsten filament through which the electric current was supplied. Automatic voltage control allowed maintaining a quasi-constant *T*_*w*_ during the whole experiment. The wall temperature *T*_*w*_ (Fig. 1(b)) under the liquid layer was constant during the entire experiment with accuracy within 0.5–1 °C. All experiments were carried for horizontal layers, when the height of the solution layer decreased continuously due to evaporation. The experimental setup along with salt solution was placed on a precise scale (2), which allowed measuring the solution mass change over time. In the vicinity of the metal wall surface 5 thermocouples (6) were located (in the center and at a distance of half the radius from the center), which allowed determining the average temperature of the wall under the liquid. Thermocouples were located close to the wall surface (at a distance of 0.1–0.3 mm). The temperature (*T*_*s*_) of the liquid interface was measured by the thermal imager (NEC R500, Fig. 1(b) (5). The resolution of infrared camera of the thermal image was 620 × 510 pixels (the spectral bandwidth of 3–5 µm). Pre-conducted test experiments have shown that the influence of the liquid layer thickness and concentration on the process of thermal imager measurement can be neglected. To measure the initial mass concentration of salt (*С*_01_), the densimeters were used. The current salt concentration was measured by the gravimetric method.

Current concentrations of salts *С*_1_ were determined using continuous measurement of the solution mass. Since the salt mass stays constant during the entire experiment, we can measure the current concentration of salt *С*_1_. As С_1_ grows with time, the mass fraction of water (*С*_2_) decreases, *C*_2_ = ${C}_{{H}_{2}O}$; where ${C}_{{H}_{2}O}={m}_{{H}_{2}O}/m$; *C*_1_ = *C*_*sol*_ = *m*_*sol*_/*m*; ${m}_{{H}_{2}O}$ is water mass; *m*_*sol*_ is salt mass; *m* (*m* = ${m}_{{H}_{2}O}$ + *m*_*sol*_) is aqueous salt solution mass. Figure 1(b) shows the profiles of temperature, salt concentration and velocity inside the liquid layer. Due to evaporation, the liquid surface is cooled, and the temperature *T*_*s*_ is minimal. The concentration of salt *C*_1*s*_ has a maximum value in the vicinity of the free surface of the solution. The temperature gradients inside the layer and on its surface are assumed to lead to the circulation of the velocity *u* inside the liquid. On the surface of the solution, there are the thermal Marangoni flow (*Ma*_*T*_) and the solutal Marangoni flow (*Ma*_*C*_).

The growth of *C*_1_ leads to a change in *p*_*s*_ at the liquid interface^2,19,21^. At the known values of *T*_s_ and *C*_1s_, the equilibrium value of *p*_*s*_ is determined by the equilibrium curve of the salt solution. The maximum relative error of *b* (kg/(cm^2^)) was 10–12%, when there was low value of evaporation rate (*b*) (for the highest salt concentration before crystallization beginning). The maximal measurement error of heat transfer coefficient *α* was 20–25% and agreed with the highest salt concentration. Under these conditions, there were minimum evaporation rates and minimum differences between the wall temperature and the free surface temperature of the liquid.

All experiments were carried out prior to crystallization. The beginning of crystallization was recorded by video camera (7) (Fig. 1(a)). The resulting salt crystals (crystalline hydrates) were clearly visible in the photos.

## Evaporation and Heat Transfer

### Experimental data

The evaporation rate *b* was determined as *b* = *∆m*/(*∆tF*)(kg/(cm^2^)) for time intervals ∆*t* = 10 s (*m* is the current liquid mass, *F* is the area of the liquid interface. Experimental data on *b* are shown in Fig. 1(c). The curves show intervals of measurement errors (**I**). The specified interval of measurement errors is maximal. The error for other points is less. The initial salt concentration for all solutions *С*_01_ = 10%. Some differences for the experimental curves in Fig. 1(c) should be noted. The rate of evaporation for salt solutions (CaCl_2_, MgCl_2_, LiCl and LiBr) decreases significantly for *t* > 500–800 s (the second salt group) and *b* for aqueous salt solutions (NaCl, BaCl_2_ and CsCl) varies weakly in a wide range of time and salt concentration (the first salts group). The evaporation rate (b) of water layer up to *t* = 700–800 s is almost constant and sharply increases further.

Let us consider the physical properties of salts, which lead to different behavior during evaporation. Evaporation rate *j* (kg/s) of a liquid layer depends on difference Δ*p*_*vs*_ (Δ*p*_*vs*_ = *p*_*vs*_ – *p*_*v∞*_), *p*_*vs*_ is the water vapor pressure, *p*_*v∞*_ is the vapor pressure of the ambient air. The evaporation rate can be presented in the form (1)^24^

1 $$j \sim D({\rho }_{vs}+{\rho }_{v\infty })\mathrm{ln}(1+{B}_{M}) \sim {p}_{vs}({\rm{if}}\,{\rho }_{vs}\gg {\rho }_{v\infty })$$

where vapor pressure *p*_*vs*_ can be expressed through density (vapor concentration) *ρ*_*vs*_ in accordance with Mendeleev-Clapeyron equation, since the vapor-air mixture under the atmospheric pressure is considered the ideal gas (coefficient *D* is the diffusion coefficient for gas-vapor mixture, parameter *B*_*M*_ is the Spalding mass number^24^). The significant growth in the salt concentration results in a decline in *p*_*vs*_. Equilibrium curves of different salt solutions vary significantly. The Table shows data for *p*_*vs*_^19–21^.

As can be seen from Table 1, for the time interval *t* = 100–500 s, values of *p*_*vs*_ for salts of the first and second groups differ slightly, which corresponds to the behavior of curves in Fig. 1(c) (*j* ≈ const (kg/s), *b* ≈ const (kg/(cm^2^))). The exception is LiCl salt. For the salt solution (LiCl/H_2_O) there is a noticeable decrease in the evaporation rate *j* and a drop in *p*_*vs*_ during the entire evaporation period. For *t* > 1000 s, the significant growth of the mass concentration of salt leads to the abrupt decrease in *j* for the second salt type, since the *p*_*vs*_ decreases markedly. However, the salts of the first group for *t* > 1000 s keep a quasi-constant value of *j*, since *p*_*vs*_ decreases rather weakly. In fact, the evaporation rate should also decrease slightly (by about 5–7%), which is below the measurement error. For water at the final stage of evaporation, there is a characteristic noticeable increase in *p*_*vs*_, which is due to an increase in the temperature of the free water surface *T*_*s*_ by 5–8 °C and a sharp decrease in the layer height. Reducing the liquid layer height several times leads to a decrease in the temperature difference ∆*T*_*s*_ = *T*_*w*_−*T*_*s*_. It is also easy to understand the reason of a higher evaporation rate of LiCl/H_2_O solution for *t* > 1300–1500 s compared with LiBr/H_2_O and CaCl_2_/H_2_O solutions. Since for a long time the aqueous solution of LiCl salt evaporated much slower than the other two solutions, then the concentration of LiCl salt becomes noticeably lower, and the pressure *p*_*vs*_ will, on the contrary, be higher.

**Table 1:** The equilibrium vapor pressure *p*_*vs*_.

group	salt	*p*_*vs*_, bar (*t* = 100 s)	*p*_*vs*_, bar (*t* = 500 s)	*p*_*vs*_, bar (*t* = 700 s)	*p*_*vs*_, bar (*t* = 1100 s)
II	LiBr	0.25	0.24	0.21	0.17
II	CaCl_2_	0.24	0.23	0.22	0.18
II	LiCl	0.23	0.19	0.15	0.08
II	MgCl_2_	0.235	0.21	0.17	0.12
I	NaCl	0.25	0.245	0.24	0.235
I	CsCl	0.25	0.245	0.24	0.235
I	BaCl_2_	0.245	0.24	0.23	0.23
	H_2_O	0.27	0.27	0.29	0.38

Let us construct the expression for the equality of heat fluxes for the interface (wall-liquid) and interface (liquid-gas). The heat flux in the solid wall (*q*_*w*_) is equal to the heat flux in liquid (*q*_*l*_). The heat entering the liquid interface is equal to the sum of evaporation heats (*q*_*e*_), convection (*q*_*c*_), radiation (*q*_*r*_) and dilution heat (*q*_*d*_) (at evaporates of salt solution) (2),

2 $${q}_{w}={q}_{l}={q}_{e}+{q}_{c}+{q}_{r}+{q}_{d}$$

where *q*_*l*_ = *α*_*l*_(*T*_*w*_−*T*_s_), *q*_*e*_ = *rj*_e_ (evaporation rate *j*_e_ = ∆*m*/*F*∆*t*), *q*_*c*_ = *α*_*g*_(*T*_*s*_-*T*_*a*_), *q*_*r*_ = *εσ*(T_s_^4^−T_*a*_^4^)^25^, the dilution heat is determined by the reference data^19–21^, *F* is the area of free surface of liquid (liquid-gas), *m* is the liquid mass, *t* is the time, *r* is the latent evaporation heat, *α*_*g*_ is the gas heat transfer coefficient, *α*_*l*_ is the liquid heat transfer coefficient, *T*_*a*_ is the temperature of ambient air, *T*_*w*_ is the temperature of metal wall surface, *T*_*s*_ is the interface temperature (liquid-gas), *σ* is the Stefan-Boltzmann constant.

According to (2), coefficient *α*_*l*_ was determined experimentally by (3).

3 $${\alpha }_{l}={q}_{l}/({T}_{w}-{T}_{s})$$

Thus, during the experiment, temperature *T*_*s*_ and temperature difference on the liquid surface Δ*T*_*s*_ varied insignificantly, and we could assume *α*_*g*_ = const with an error below 2%. Coefficient *α*_*g*_ for the gas phase was calculated by expression connecting the Rayleigh (*Ra*) and Nusselt (*Nu*) numbers (*Nu* = *α*_*g*_*R*/*λ*_*g*_ = 0.54*Ra*^0.25^^25^, where *R* is the radius of liquid surface, *λ*_*g*_ is the gas thermal conductivity, *Ra* = *gβ*Δ*T*_*s*_(*R*)^3^/*νa*, *β* is the thermal expansion coefficient, *g* is the gravity acceleration).

**Figure 2:**
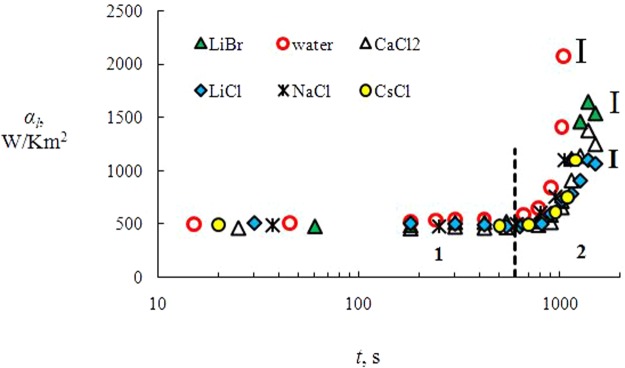
A change in coefficient *α*_*l*_ with time (*С*_01_ = 10%; *T*_w_ = 80 °C); 1 and 2 are the stages of heat transfer.

Experimental curves for coefficient *α*_*l*_ for solutions of salts (LiBr, CaCl_2_, NaCl, CsCl, LiCl) and water are presented in Fig. 2. The graph shows the confidence intervals. For curves *α*_*l*_, we can obviously distinguish two regimes of heat exchange: (1) weak growth of *α*_*l*_ (*t* < 700 s for water, and *t* < 800–900 s for salt solutions); (2) substantial increase in *α*_*l*_ for *t* > 900 s (water) and *t* > 1000 s for salts. The value of extremum *α*_*l*_ for aqueous salt solutions is inversely proportional to layer height *δ* (*α*_*l*_ = *λ*/*δ)*. The lowest value for height *δ* relates to LiBr salt. For the CaCl_2_ salt, the height *δ* is lower, and for the LiCl salt *δ* is the minimum value (this salt had a minimum mean evaporation rate for most of the evaporation time). For time *t* > 1200 s, the conductive heat transfer has a predominant effect on heat and mass transfer and evaporation. In addition, there may be a higher Δ*C*_s_ value for the aqueous solution of LiBr, and thus a higher value of *Ma*_*C*_ (the solutal Marangoni number) than for the other salts.

**Figure 3:**
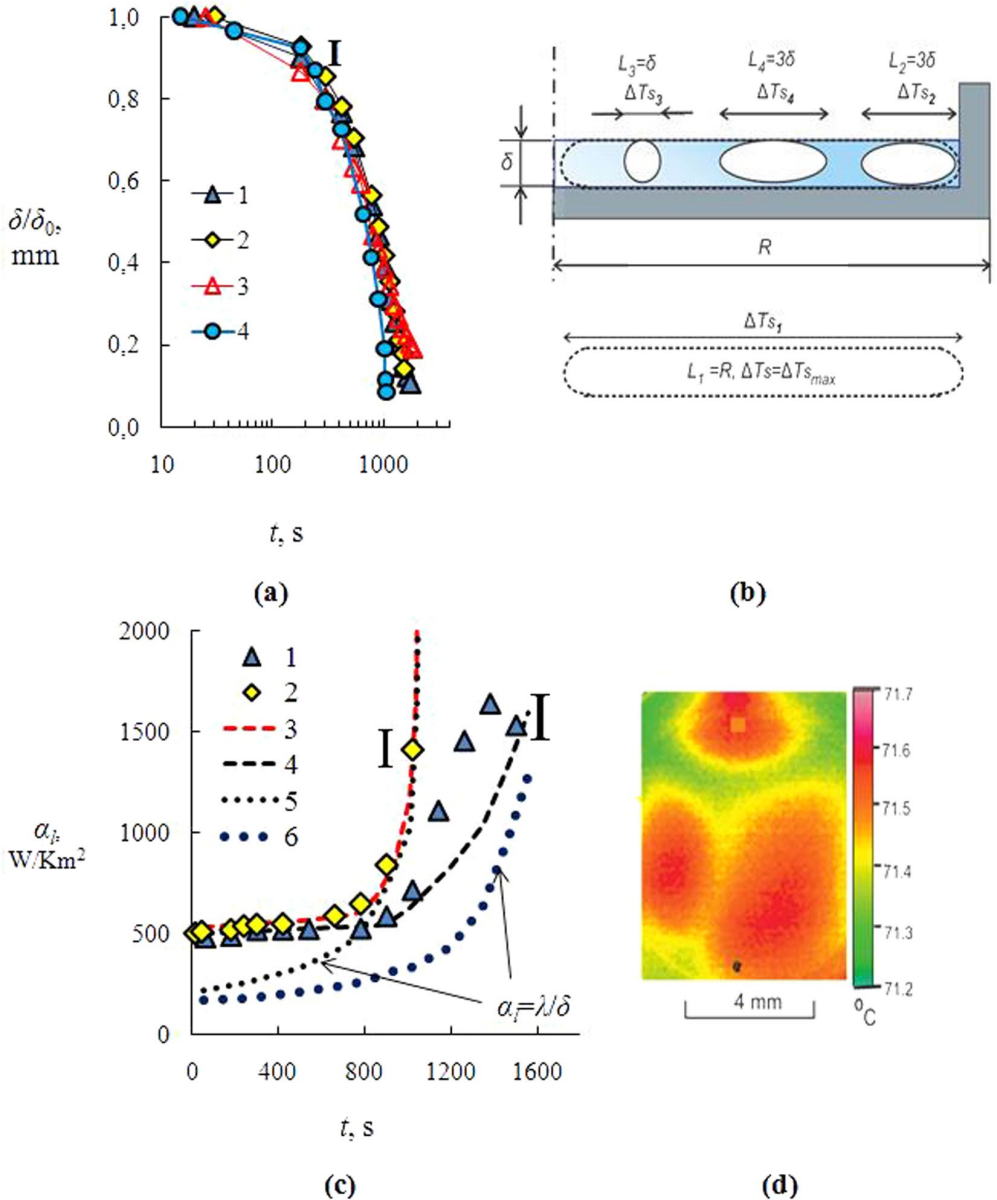
(**a**) Change in *δ*/*δ*_0_ versus time (*δ* is the layer height; *δ*_0_ is the initial layer height; *С*_01_ = 10%; *T*_w_ = 80 °C): 1 – LiBr; 2 – CaCl_2_; 3 – LiCl; 4 – H_2_O. (**b**) Characteristic vortices in a liquid film with longitudinal size *L*_1_– *L*_4_; (**c**) changes in the heat transfer coefficient *α*_*l*_ over time (С_01_ = 10%; *T*_w_ = 80 °C; 1, 2 – experimental data, 3, 4 – calculation (*L*_4_ = 3*δ*): 1, 4 – LiBr; 2, 3 – H_2_O; curves 5, 6 – *α*_*l*_ = *λ*/*δ* (conductive heat transfer). **I** is the interval of measurement errors; (**d**) Thermal image at multiple magnification (water, *t* = 500 s, *δ* = 1.5–2 mm).

The role of convection and heat conductivity in heat exchange depends on layer height *δ*. With a decreasing layer height, the effect of free convection in liquid decreases (the values of *Ra* and *Ma* decrease). Figure 3(a) shows experimental data of the liquid layer *δ*/*δ*_0_ (*δ*_0_ is the initial layer height) for H_2_O and solutions of salts: LiBr/H_2_O, CaCl_2_/H_2_O, LiCl/H_2_O. Figure 3(a) shows data only for three salts to avoid overloading the graphs with information, i.e., to make the points distinguishable. The behavior of MaCl_2_ from the second group is qualitatively consistent with the behavior of LiBr (CaCl_2_). The behavior of salts of the first group corresponds to water.

### Analysis of experimental data

Most of the existing simplified analytical models do not take into account the circulation inside the liquid layer due to the buoyancy and Marangoni convection. These approximations can lead to a significant understatement of heat exchange. Recently three-dimensional flows of water^26^ and alcohol solution^27^ have been considered with the help of direct numerical simulation. However, the lack of experimental data does not allow judging on the accuracy of these calculations. In addition, this simulation deals with evaporation on the substrate without a high heat flux, which significantly differs from the conditions of this study (high-temperature evaporation). As stated in the Introduction, the behavior of salts is fundamentally different from multicomponent solutions, when all components are highly volatile liquids.

For a qualitative description of the aqueous salt solution, a simplified model is proposed. It is based on the simultaneous consideration of convection and conduction in the heat exchange. It is assumed that the laws of change in the height of the layer *δ* and ∆*T*_*w*_ are known. In the calculation, *δ* and ∆*T*_*w*_ are taken on the basis of experimental data. To determine *δ* by calculation, it is necessary to solve the equation for the evaporation rate *j*, which is the subject of further research. In addition, the determination of *j* is not trivial either, since a long layer and high buoyancy of air will lead to a significant intensification of evaporation and heat exchange (the effect of free convection in the gas phase). The purpose of the subsequent analysis is to show the need to take into account the influence of convection on heat exchange in a thin layer and to determine the characteristic convective scales, which is extremely important for a fundamental understanding of this problem and for calculations of numerous related problems for heat exchange of drops, jets, and films.

The linear relation of velocity with the Marangoni number was first proposed in^5^. The velocity *u* in a liquid layer is considered as a sum of velocities of key components *u* = *u*_*Ra*_ + *u*_*MT*_ + *u*_*MC*_ + *u*_*a*_, where *u*_*Ra*_ = *k*_*T*_*Ra* (influence of *Ra* number), *u*_*a*_ = *a*/*δ* (conventional velocity, characterizing the conductive heat transfer), *u*_*MT*_ = *k*_*T*_*Ma*_*T*_ (influence of the thermal Marangoni number), *u*_*MC*_ = *k*_*C*_*Ma*_*C*_, (influence of the solutal Marangoni number). The thermal Marangoni number *Ma*_*T*_ = (Δ*T*_*s*_*L*_*TL*_/*μa*)·(*dσ*/*dT*_*s*_), and the solutal Marangoni number *Ma*_*C*_ = (Δ*C*_*s*_*L*_*TL*_/*μD*)·(*dσ*/*dC*_*s*_), where Δ*C*_*s*_ is the difference in the concentration of salt at the liquid interface, and *D* is the coefficient of diffusion in the salt solution, *μ* is the viscosity of liquid, *a* is the liquid thermal conductivity, *σ* is the liquid surface tension, *δ* is the layer height, *L*_*TL*_ is characteristic size. According to estimates, at the layer height of less than 3 mm the Raleigh number influence of less than 10% and buoyancy in the liquid can be neglected. Then, the Peclet number *Pe* may be written in the form *Pe* = (*u*_*MT*_ + *u*_*MC*_ + *u*_*a*_)/*u*_*a*_ = (*Pe*_*С*_ + 1), *u*_*C*_ = *u*_*MT*_ + *u*_*MC*_ (velocity in the liquid due to Marangoni convection), *Pe*_*С*_ = (*u*_*MT*_ + *u*_*MC*_)/*u*_*a*_ is the convective Peclet number. As a result of generalization of experimental and calculated data^5,28–30^, *k*_*C*_ = 2.96·10^–10^ (m/s), *k*_*T*_ = 0.15·10^−7^(m/s). In such formulation, there is no sense in neglecting the inertial terms and considering only the creeping flow, since in the limiting cases we have to obtain a purely conductive heat transfer, when *Pe*_*С*_ = 0 (*u*_*C*_ = 0), and vice versa, when *Pe*_*С*_ ≫ 1, the number of *Pe*_*С*_ shall turn into *Re* number retaining the power dependence for the laminar flow regime (*n* = 0.5). Then, the Nusselt number (*Nu* = *α*_*l*_*δ*/*λ*, *λ* is the thermal conductivity of liquid) and the heat transfer coefficient *α*_*l*_ in a liquid layer may be written as expressions (4).

4 $$Nu={(P{e}_{C}+1)}^{0.5},\,{\alpha }_{l}=\lambda /\delta {(P{e}_{C}+1)}^{0.5}$$

In order to calculate the expression (4), experimental data on the velocity profile inside the drop are necessary that will become the subject of further research. To date, there is very little data on velocity measurements in thin films and small droplets, evaporating on a wall whose temperature is 80 °C or higher. With increasing wall temperature and at high temperature gradients inside the liquid, the measurement error increases. In addition, there is a significant non-stationarity of the velocity field and temperature inside the liquid, which complicates the determination of the average temperature and velocity for the entire height of the liquid layer. From statistical theories it is well known that the higher the period of non-stationarity, the longer the period of integration is necessary to take to determine the statistical moments. In our case, there is a wide range of pulsations, and the unsteady temperature surges of the highest wavelength (defined by the thermal imager) are the order of 5–20 s. Then, the time averaging has to exceed 100–1000 s. This averaging is impossible, since the layer height *δ* changes fast, and the velocity field changes (the velocity decreases with decreasing *δ*). Reducing the averaging time by spatial averaging is impossible because there is no region with a uniform velocity profile in the layer. There is one more difficulty in statistical processing. The random variable distribution function will not be described by the Gaussian function, i.e. the random variable distribution function is unknown. However, the expression (4) can be used quite effectively for qualitative analysis even without measuring the profile of velocity *u* inside the liquid, since *u* is expressed through the Marangoni number.

Further, the analysis will use the data of thermal imager measurements of temperature inhomogeneities on the liquid layer surface. Features of temperature measurement in thin layers and solution drops with the use of a thermal imager are considered in^31,32^. Figure 3(d) shows the picture (obtained by the thermal imager) of the layer surface in the area in the layer center with multiple magnification. One can clearly see the characteristic temperature inhomogeneity (temperature scales with longitudinal size *L* = 4–7 mm). The temperature difference ∆*T*_*s*_ along these temperature inhomogeneities on the liquid surface is 0.2–0.7 °C. The layer height *δ* was approximately 2.5 mm. Thus, the maximum size of *L* exceeded the value of *δ* about 3 times. These values will be further needed to determine the *Ra* and *Ma* numbers.

Values of *Ma* number will depend on the characteristic scale of *L*. Temperature difference Δ*T*_*s*_ on the free liquid surface also depends on *L* and distribution of *T*_*s*_ is known from thermal imager measurements. Thus, it is sufficient to consider several characteristic scales, the size of which is associated with a certain physical sense. Figure 3(b) contains four scales of *L*, related to the following hypotheses: (1) *L*_1_ equals the surface radius (*L*_1_ = *R*). In this case, it is assumed that the longitudinal scale of the vortices is 10–30 times higher than the transverse one. This circulation is possible if the vortices are not broken down into smaller ones, i.e. they are resistant to disturbances. (2) *L*_2_ is located in the vicinity of the side wall (*L*_2_ = *3δ*). Then Δ*T*_*s*_ = *T*_*w*_−*T*_*s*_ = 10–12 °C (at *t* = 100–700 s), where *T*_*w*_ = 80 °C is the temperature of the side wall, which coincides with the temperature of the horizontal wall of the heater. (3) Vortices are maximally unstable to perturbations and their longitudinal size coincides with the transverse one (the shape of the sphere). There is a three-dimensional rotation inside the sphere. Then the whole fluid layer consists of a sequence of spheres within the layer. The number of spheres over the length of the heater radius is equal to *R*/*δ*. At *L*_3_ = *δ*, the temperature difference Δ*T*_*s*_ = *T*_*s*(max)_−*T*_*s*(min)_ = 0.22 °C. (4) According to thermal imager measurements (Fig. 3(d), the specific size of temperature inhomogeneity *L*_4_ = (3–4)*δ*. Longer vortices are unstable and break down into smaller ones. In this case Δ*T*_*s*_ = *T*_*s*(max)_−*T*_*s*(min)_ = 0.22·3 = 0.66 °C. Table (2) presents the calculation data in accordance with expression (4) and four characteristic dimensions of *L* (Fig. 3(b)). The table gives the value of heat transfer coefficient when there is only a conductive heat transfer (*u* = 0), *α*_*cond*_ = 220 W/Km^2^.

**Table 2:** The heat transfer coefficient *α*_*l*_ for the layer of water at different characteristic scales of vortices *L* in the layer and temperature difference ∆*T*_*s*_ on a free liquid surface at *t* = 150 s (*δ* – liquid layer height, *δ* = 2.5 mm, *R* – layer surface diameter, *R* = 35 mm, experimental value *α*_*exp*_ = 525 W/Km^2^, heat transfer coefficient at conductive heat transfer *α*_*cond*_ = 220 W/Km^2^).

Characteristic scale, mm ∆*T*_*S*_, °C	*L*_1_ = *R*, 3 °C	*L*_2_ = *3δ*, 10 °C	*L*_3_ = *δ*, 0.22 °C	*L*_4_ = *3δ*, 0.66 °C	*L*_4_ + *L*_2_,
***α***_***l***_ **, W/Km** ^**2**^	1320	1120	270	390	536

According to the table, scales *L*_1_ and *L*_2_ result in multiple overestimation of heat transfer compared with the experiment. Vortices with dimensions *L*_3_ = *δ*, on the contrary, show two times underestimation of heat transfer. Calculation using the thermal scale *L*_4_ gives an understated value of *α*_*l*_. Thus, none of these scales allows generalizing the experiment. These conclusions are quite obvious, as there are at least two characteristic scales *L*_2_ and *L*_4_. In the conditions of the considered experiment it is impossible to ignore the influence of the side walls, which have a significant impact on heat transfer. Then the problem has to consider the joint impact of different vortices, located along the horizontal line. It is necessary to normalize the heat transfer of individual vortices in accordance with the expression (*α*_*l*2_*f* )*n*_2_ + (*α*_*l4*_*f* )*n*_4_ = (390·0.2)·4 + (1120·0.2)·1 = 536 W/Km^2^. At that the condition of normalization is performed: *fn*_2 + _*fn*_4_ = 1, where *f* = 0.2 (normalization coefficient), *n* is the number of vortices, *n*_2_ = 1, and *n*_4_ = 4. Along the layer radius *R* there are about 5 vortices of one size with a longitudinal length of each vortex equal to 3*δ*. The obtained value of heat transfer coefficient coincides with the experiment (525 W/Km^2^). Thus, the longitudinal length of the vortex *L*_2_ = *L*_4_ = 3*δ* allows describing the experiment (Table 2) and coincides with thermal imager measurements (Fig. 3(d)).

From the conducted analysis one more important conclusion may be drawn. The heat exchange in the layer strongly depends on the ratio *δ/R*. Two limiting cases may be considered: (1) If *δ* = *R*, the temperature difference on the layer surface will increase many times, since Δ*T*_*s*_ = Δ*T*_*s*2_ = *T*_*w*_−*T*_*s*min_, where *T*_*w*_ is the temperature of the hot side wall. (2) If *R* ≫ *δ*, then the influence of the side wall (the influence of the scale *L*_*2*_) can be neglected and the heat transfer in this case will decrease compared to the case of joint influence, when two characteristic scales are to be taken into account. It should be noted that the scales *L* = *L*(*t*) do not have constant size, but decrease with time, since they are related to the layer height *δ* (*L* = 3*δ* is the condition of hydrodynamic stability), which decreases over time due to evaporation. Reducing *L* over time leads to a decrease in the role of convection. When *δ* tends to zero, the contribution of convection also asymptotically tends to zero.

The last sentence remains in question. The influence of the main characteristic vortices (predominant influence) has been discussed above. However, at the same time there should be a small contribution of the vortex *L*_1_ = *R*. The article does not consider the statistics of the occurrence of vortices of different sizes. It is obvious that in reality there is a continuous spectrum of scales and spectrum of energies from the wave number. In a general form, it is necessary to consider the probability function. Since it is impossible to choose the necessary period of averaging of statistical moments in this non-stationary flow, it is also extremely difficult to experimentally determine the type of probability functions. Further development of this technique is also associated with the use of probability functions. An example of this flow is the circulation near the precursor film^33^. Despite the very low evaporation rate (a water drop evaporated at room temperature of 28 °C and air humidity of 60%) in a thin film (film height *h* ≥ 0.1 µm and length *l* = 20 µm), sufficiently high velocities of 0.02 mm/s are realized in the liquid. Visualization of particle tracks using a fluorescent microscope has detected the presence of circulation flow at extremely low layer height and low temperature difference Δ*T*_*s*_ (*l*/*h* = 200). This motion in the water was caused by the Marangoni surface flow, and the long vortex did not break down into smaller ones. Probably, vortices with very low velocity (about 0.001–0.05 mm/s are more resistant to very weak external disturbances, or disturbances are suppressed at such film thickness).

Figure 3(c) shows the experimental data (curves 1 (LiBr) and 2 (H_2_O)) and the calculated curves (3 and 4) for the heat transfer coefficient in the water layer (curve 3) and the aqueous solution of LiBr (curve 4), as well as curves for purely conductive heat transfer (curves 5 and 6); the confidence intervals are specified (**I**). Figure 3(c) presents the calculation curve for LiBr salt only. Description of other salts also fairly agrees with the experimental data. Thus, it is possible to generalize different salts from the first and second groups at a wide range of changes in the layer height, concentration and viscosity. The calculated curves correspond to the combined effect of the characteristic scales *L*_2_ and *L*_4_ (calculation methodology has been discussed above). The growth of convection will lead to an increase in the heat transfer. As can be seen from the figure, the calculation for water and for an aqueous salt solution corresponds to the experiment in the whole range of the evaporation time, i.e., when *u*_*C*_ > *u*_*a*_ (small and medium times) and *u*_*a*_ > *u*_*C*_ (the final stage of evaporation), where *u*_*C*_ is the convective velocity of the fluid, and *u*_*a*_ is a conditional rate of conductive heat transfer (*u*_*a*_ = *a*/*δ*). For aqueous salt solution, for *t* > 1000 s there is a noticeable excess of the experiment over the calculation. This overstatement is probably due to the stronger influence of the solutal Marangoni flow. Since there is no data on the change of Δ*С*_*s*_ over time, the constant difference in salt concentrations Δ*С*_*s*_ = const = 8% was taken for the entire time of calculation. This value is set experimentally at the beginning of crystallization. Over time, there is a multiple increase in the solution viscosity. It is obvious that the increase in viscosity and convection suppression in the liquid will contribute to the growth of Δ*С*_*s*_. Thus, the solutal Marangoni number will increase with the increase of evaporation time and with the increase of Δ*С*_*s*_. The growth of convection will lead to an increase in heat exchange.

Thus, solutions with high viscosity and low layer height can demonstrate a significant intensification of heat transfer that must be considered in the simulation and in the management of technologies, aimed at creating surfaces with a strictly predetermined morphology of structures: deposition of colloidal particles, plasma spraying of nano-coatings, crystallization from the melt, jet cooling of metals, etc.

The proposed method of normalization allows taking into account the statistical influence (statistical weight) of each individual vortex. For the development of this technique in difficult conditions of inhomogeneous temperature and velocity fields, there is a need in measurements of instantaneous velocity and temperature fields using the MicroPIV and PLIF methods.

## Conclusion

Experimental studies of aqueous solutions of salts LiBr, LiCl, CaCl_2_, NaCl, MgCl_2_, BaCl_2_, CsCl have been carried out, and the method of heat exchange calculation for a layer of water and aqueous solution of salt has been presented.

I suggest a simple qualitative method for estimating the heat transfer coefficient in a thin layer of solution using several characteristic scale of circulation. Each scale has a certain physical sense and it is conditioned by the Maragoni flow. This is scientific novelty of research.

Experimental data on heat transfer and evaporation have shown that salt solutions can be divided into two types: (1) For the first type of salt solutions (CsCl, BaCl_2_ and NaCl), evaporation rate *j* is quasi-constant; (2) The second type of salts (LiCl, LiBr, MgCl_2_ and CaCl_2_) is characterized by a drastic decrease in *j* with an increase in mass concentration of salts, caused by a drastic decrease in the equilibrium partial pressure of water vapor on the free surface of a liquid layer.

Under the natural conditions and in technology, salt solutions play an important role. There is a great variety of salt solutions, and criteria for selecting the methods of calculation are necessary. This paper shows that evaporation and heat transfer of salts are significantly different for the first and second types. The difference in the behavior of salts of the first and second types can be explained by the expression for the evaporation rate and partial vapor pressure *p*_*vs*_, which are determined with the help of the known equilibrium curves. For salts of the second type, *p*_*vs*_, decreases many times with increasing salt concentration, and for the salts of the first type, the partial vapor pressure decreases slightly over evaporation time. For this type of salts, there is a low degree of solubility, if the volume concentration of salt rather than the mass one is determined. Thus, for salts of the first type we can consider the simplified modeling with quasi-isothermal approximation and *j* = const.

The thermal Marangoni flow should be taken into account when calculating heat exchange in a thin water layer. The thermal and solutal Marangoni numbers should be taken into account to calculate coefficient *α*_*l*_ for liquid solutions. The neglect of *Ma* leads to a twofold underestimation of heat transfer for water and its three to four - fold underestimation for the salt solution. Heat exchange curves for both H_2_O and salt solutions have a strongly nonlinear form, so it is incorrect to take *α*_*l*_ = const, as it is done in most studies.

The modeling of heat transfer is correct only if the correct determinant size of vortices in the solution layer is chosen. The conducted thermal imaging measurements have shown that the longitudinal size of stable vortices is 3–4 times higher than the height of the layer *δ*. The simulation with four characteristic sizes of *L* has been performed. The simulation results show that it is the established size *L*_4_ = 3*δ* that allows describing the experimental data.

Experimental and calculated data show that it is necessary to take into account the combined effect of two characteristic scales, i.e. heat and mass transfer in the vicinity and at a distance from the side wall. In the limiting cases *δ* = *R* and *R* ≫ *δ* the calculation is made with regard to only one characteristic longitudinal size of the vortex. In real conditions, there is often a side wall with variable temperature, and temperature change along the layer is realized. When crystallizing in the layer, there are large temperature gradients not only in the transverse but also in the longitudinal direction. These thermal gradients lead to the presence of multiple characteristic sizes of vortices that need to be considered in the simulation.

For the first time, a simple technique is proposed to satisfactorily describe the heat exchange both for the case *u*_*C*_ > *u*_*a*_ (excess of convection over conduction) and for *u*_*C*_ < *u*_*a*_. This takes into account both the conductive heat transfer and the thermal and the solutal Marangoni numbers. Usually the influence of the Marangoni flow is neglected, considering that the surfactant suppresses convection. In fact, the convection suppression is possible, but not in full; i.e. convection makes a significant contribution to the heat exchange of the aqueous salt solution even at very low layer height (0.3–0.5 mm) and at very high viscosity, which corresponds to the aqueous solution of LiBr near the crystallization point.

It should be noted that in case of multiple reduction of the diameter of the solution layer surface, the contribution of the other characteristic scales will change as well. In this case, the scale *L*_2_ will be decisive. As a result, the Δ*С*_*s*_ and Δ*T*_*s*_ will increase several times due to the influence of the wall, which will lead to the increased convection and heat transfer compared to the variant considered in this article.

Thus, experimental data and simulation results of this article contribute to further research aimed at measuring the instantaneous velocity and temperature fields inside the layer, identifying the characteristic scales of vortices, and solving problems of the stability of these vortices.
